# Retraction Note: Jawbone microenvironment promotes periodontium regeneration by regulating the function of periodontal ligament stem cells

**DOI:** 10.1038/s41598-023-36331-w

**Published:** 2023-06-20

**Authors:** Bin Zhu, Wenjia Liu, Yihan Liu, Xicong Zhao, Hao Zhang, Zhuojing Luo, Yan Jin

**Affiliations:** 1 State Key Laboratory of Military Stomatology, Centre for Tissue Engineering, School of Stomatology, Fourth Military Medical University, Xi’an, Shaanxi People’s Republic of China; 2 Department of Orthopedics Surgery, Xijing Hospital, Fourth Military Medical University, Xi’an, Shaanxi People’s Republic of China; 3 Department of Stomatology, PLA Xizang Military Region General Hospital, Lhasa, Tibet People’s Republic of China; 4 Department of Stomatology, PLA 301th Hospital, Beijing, People’s Republic of China

Retraction of: *Scientific Reports* https://doi.org/10.1038/srep40088, published online 05 January 2017

The Editors have retracted this Article.

Concerns were raised about duplication of areas in Figure 6C-3 (third panel), between Figure 2A-1-1 and Figure 2A-2-1 (first panels on top and middle rows), and between Figure S1A-1 and Figure S1A-2 (first and second panels of the top row). Analysis of data files provided by the authors has shown multiple irregularities.

The Editors therefore no longer have confidence in the data presented.

All Authors disagree with this retraction.

